# The learning primacy hypothesis of dopamine: reconsidering dopamine’s dual functions

**DOI:** 10.3389/fncel.2025.1538500

**Published:** 2025-04-15

**Authors:** Charltien Long, Sotiris C. Masmanidis

**Affiliations:** ^1^Department of Neurobiology, University of California, Los Angeles, Los Angeles, CA, United States; ^2^Medical Scientist Training Program, University of California, Los Angeles, Los Angeles, CA, United States; ^3^California Nanosystems Institute, University of California, Los Angeles, Los Angeles, CA, United States

**Keywords:** dopamine, striatum, learning, movement, electrophysiology

## Abstract

The dopaminergic modulation of striatal circuit function remains intensely studied and debated. Nevertheless, a prevalent view is that striatal dopamine serves important roles in both reinforcement learning and the performance of movements, two highly distinct processes. But this dichotomy has led to a longstanding problem of how to interpret the functional consequences of a particular dopaminergic signal—is it to learn or to move? In order to explore this ambiguity and approach a possible resolution, this review examines the key evidence for dopamine’s role in learning and movement. As part of that discussion, we consider a recent body of evidence that views the common dichotomous perspective through a more nuanced lens, by suggesting a comparatively limited dopaminergic contribution to movement. This concept, which we refer to as the learning primacy hypothesis, offers a unified conceptual framework for understanding dopaminergic function.

## Introduction

As with a dog performing a trick for a treat, many actions are learned through rewarding experiences. Such behaviors involve two distinct processes: one for learning about what the reward is associated with, and another for performing actions in pursuit or anticipation of the reward (“movement,” also referred to as performance). While learning and movement often operate synergistically, a key distinction is the behavioral timescale over which they occur. Reinforcement learning concerns the updating of what animals will do in the future. If a dog receives a treat after performing a certain trick, it is more likely to repeat the action next time it is prompted. Movement, by contrast, involves initiating or performing the trick itself, and more generally, motor responses on fast timescales of seconds or less. One of the most fascinating—and puzzling—features of midbrain dopaminergic neurons is that they appear capable of impacting both learning and movement through their actions in the striatum. This dual behavioral role is supported by a wide body of literature, but also presents a substantial challenge that has long vexed the field: the ambiguity in interpreting the functional consequence of a particular dopaminergic signal. Is it to learn, move, or both (Berke, 2018; Berridge and Robinson, 1998; Wise, 2004).

**FIGURE 1:**
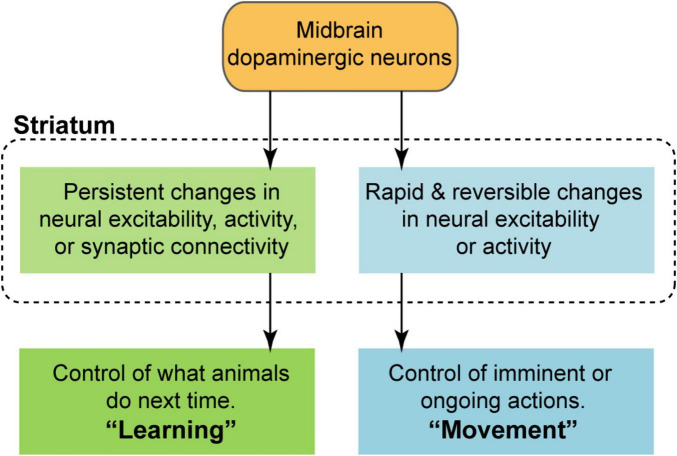
Conceptual model of the dopaminergic modulation of striatal circuit function under the framework of dual roles in learning and movement. Reinforcement learning requires the ability to induce some form of persistent change, i.e., synaptic plasticity, over slow timescales (typically greater than 1 s). Movement, by contrast, requires the ability to rapidly and reversibly alter neural excitability or activity on behaviorally relevant timescales (typically less than 1 s).

Much of the recent attention in the field has centered on what information is, or is not represented by the dynamics of dopaminergic neurons and their striatal projections. As valuable as this work is, on its own it cannot provide a complete picture of dopaminergic circuit function. This is because we do not yet have a clear understanding of how these dopaminergic signals act on the striatum to influence behavior (Bamford et al., 2018; Calabresi et al., 2007; Gerfen and Surmeier, 2011; Kreitzer and Malenka, 2008; Nicola et al., 2000; Sippy and Tritsch, 2023). The distinct timescales involved in learning and movement (as defined above) imply different neural mechanisms are at play. On the one hand, learning requires some form of persistent change in neural activity, e.g., via synaptic plasticity. On the other hand, movement depends on the ability to rapidly and reversibly alter neural activity, e.g., via a fast-acting neurotransmitter. Thus, to take the view that dopaminergic neurons effectively serve both of these roles, one must assume that two distinct modulatory effects in the striatum are possible (Figure 1): (1) the induction of persistent changes in activity, excitability, or synaptic connectivity in order to mediate learning, and (2) rapid and reversible changes on behaviorally relevant timescales in order to facilitate movement. Crucially, learning and movement unfold over different timescales—learning is slower, and movement is faster (for the purposes of this review, slow is operationally defined as greater than 1 s, and fast is defined as less than 1 s).

Despite decades of work, there is lasting uncertainty regarding the mechanisms and strength of dopaminergic modulation of striatal circuits at these different timescales. To appreciate why this knowledge gap is problematic it is instructive to consider a specific case of the most well-known of all dopaminergic signaling properties, reward prediction error (RPE). Of course, since its discovery, RPE has been widely thought to serve as a teaching signal in error-based reinforcement learning (Schultz et al., 1997). But this learning-centered interpretation implicitly assumes that dopamine RPE signals are tied to some form of plasticity. A completely different, movement-centered interpretation would follow if the same signals were instead tied to rapid changes in downstream activity, as in fact implicitly suggested by one study (Bakhurin et al., 2023). By the same logic, one cannot take for granted that dopaminergic signals which are correlated to specific motor parameters are actually guiding the production of those movements on a moment-by-moment basis. Depending on how these signals impact striatal circuits, they may instead be involved in reinforcing certain actions even without explicit rewards (Markowitz et al., 2023). These examples highlight the potential uncertainty in interpreting the functional significance of dopaminergic dynamics if their modulatory effects in areas such as the striatum are not well-characterized. Clarifying these modulatory effects is likely critical to better identifying mechanisms and treatments for a variety of brain disorders involving aberrant dopaminergic signaling, including Parkinson’s disease, addiction, obsessive compulsive disorder, attention deficit hyperactivity disorder, schizophrenia, and depression (Krause et al., 2000; McCutcheon et al., 2019; Nutt et al., 2015; Seiler et al., 2022; Tye et al., 2013; Zhai et al., 2019).

This review considers the key behavioral and physiological evidence supporting the view that dopaminergic neurons play an important role in both learning and movement. We attempt to add nuance to the standard position (the dual role model) by suggesting that, while the dopaminergic control of learning and movement are both possible, dopaminergic neurons are not equally important for these processes. Specifically, we bring together findings from recent work pointing to limited behavioral and electrophysiological effects of physiological dopaminergic signaling on fast timescales, and a stronger contribution of dopaminergic neurons to reinforcement learning. We refer to this concept as the learning primacy hypothesis of dopamine, though we are not the first to propose this idea (Coddington and Dudman, 2019). In addition to considering the supporting evidence we discuss some potential objections to this hypothesis as well as its implications.

## Evidence for dopaminergic neurons’ role in learning and plasticity

The contribution of dopaminergic neurons to learning is a longstanding idea whose history can arguably be traced back as far as the classic 1954 paper from Olds and Milner (1954). They found that there were several brain regions whose electrical stimulation produced behavioral reinforcement, implying that not only was reinforcement neuroanatomically accessible but also that it was dependent on a distributed network of brain areas. It was later appreciated that the reinforcing effects of this self-stimulation and other learning paradigms could also be disrupted by blocking dopaminergic neurotransmission, either pharmacologically or surgically through lesions (Fibiger et al., 1987; Fouriezos and Wise, 1976; Le Moal and Simon, 1991; Wise, 2004). It was also discovered that drugs of abuse caused the release of dopamine, and in a sense drove learning in the form of addiction (Di Chiara, 1995; Koob, 1992; Wise and Hoffman, 1992). These early discoveries cemented a role for dopamine in learning, but they were limited by a lack of specificity in the cells or pathways targeted, with experiments involving pharmacological interventions further limited to long timescale effects.

Much of the field’s current conceptualizations about dopamine’s role in learning descend from a seminal paper from Schultz et al. (1997) that described a phenomenon that would come to be known as RPE, reflecting the difference between the predicted and actual value of rewards at each point in time. However, on their own these measurements cannot establish a causal role for RPE signals in driving learning. Optogenetics has provided some of the strongest causal evidence for dopamine’s role in learning due in large part to this technique’s ability to selectively target dopaminergic neurons in a temporally precise manner. One of the first examples of this came from Tsai et al. (2009), who showed that optogenetic activation of dopaminergic neurons was able to produce conditioned place preference. Later, Steinberg et al. (2013) tested a behavioral effect predicted by RPE, by showing that optogenetic stimulation of dopaminergic neurons was able to induce learning of the novel component of a compound cue in a blocking paradigm. They also found that stimulation was able to slow the rate of behavioral extinction for reward-seeking when a sucrose reinforcer was substituted for water or omitted completely. A third study conducted by Chang et al. (2016) showed that inhibition of dopaminergic neurons at the time of rewards led to behavioral changes in a Pavlovian over-expectation task that were consistent with the predictions of RPE.

Complementing this behavioral evidence is a series of investigations demonstrating that dopaminergic signaling is capable of inducing synaptic plasticity within the striatum—a key ingredient for learning (Figure 1). A foundational set of experiments conducted by Reynolds et al. (2001) found that intracranial electrical self-stimulation of the substantia nigra potentiated corticostriatal synapses, and that this effect depended on dopaminergic receptor signaling. This study was a major step forward because it identified neuroanatomical targets and neurobiological mechanisms involved in reinforcement learning, effectively bridging behavioral and cellular neuroscience. Later work from Shen et al. (2008) confirmed that D1 and D2 receptor expressing striatal medium spiny projection neurons (MSNs) can experience dopamine-dependent long term potentiation and depression, and that this process is disrupted in a dopamine depleted state. The synaptic and molecular mechanisms for dopamine-driven plasticity were further explored by Yagishita et al. (2014). They found that stimulating dopamine after glutamate release promoted dendritic spine enlargement and protein kinase A (PKA) activation in D1 MSNs. Interestingly, these effects only occurred if the timing of the inputs was properly coordinated, requiring that dopamine follow the glutamatergic inputs within a narrow interval not exceeding 2 s; in other words, they revealed a spike timing dependent plasticity (STDP) mechanism gated by dopaminergic signaling. Stimulating dopamine release before, or more than 2 s after glutamate release led to no dendritic spine enlargement.

The induction of synaptic plasticity is thought to be accompanied by altered neural dynamics and information processing within the striatum, and recent work has begun to investigate these dopamine-dependent changes *in vivo*. Oettl et al. (2020) showed that optogenetically evoked dopamine release paired with an odor stimulus is sufficient to selectively reinforce the neural representation of that odor in the ventral striatum. This result appears analogous to how associative learning selectively enhances striatal responses to reward-paired but not unpaired odor stimuli as shown previously (Bakhurin et al., 2016). Oettl et al.’s (2020) work was important for providing direct electrophysiological evidence that phasic dopaminergic activity can mimic the effect of natural rewards on striatal dynamics, in agreement with behavioral experiments (Saunders et al., 2018; Steinberg et al., 2013). Together, these are among key studies that have helped to establish a role for dopaminergic neurons in associative learning and striatal plasticity.

## Evidence for dopaminergic neurons’ role in movement

While our discussion thus far has focused on the historical evidence behind dopamine’s role in learning, there was an equally prevalent track of interest in dopamine’s role in movement. Arguably, dopamine research was catalyzed by an early appreciation of its involvement in movement, with pathologists noting the loss of nigrostriatal dopamine in Parkinson’s disease (Hornykiewicz, 2006). This led to the adoption of dopamine replacement therapy (L-DOPA) as a mainstay of Parkinsonian motor symptom treatment (Carlsson et al., 1957). Psychostimulants that alter dopaminergic neurotransmission, as well as pharmacologically induced dopaminergic lesions typically have strong effects on motor function (Le Moal and Simon, 1991). Moreover, early work examining intracranial electrical stimulation of dopaminergic pathways proposed a separate energizing effect outside of its potential for reinforcement (Wise and Rompre, 1989).

Around the same time as the discovery of RPE coding, Berridge and Robinson (1998) presented a hypothesis challenging the learning-centered perspective, by proposing that dopamine is instead involved in incentive salience (“wanting”), a process that directly leads to reward-seeking behaviors. Returning to our analogy with the dog, incentive salience places greater weight for dopamine in motivating animals to perform the trick rather than learning about its rewarding consequences. While the position that dopamine is not involved in learning seems difficult to defend given rigorous counterevidence, a more inclusive perspective may be that the incentive salience framework claims that dopamine is vital for both learning and the pursuit of rewards. RPE signaling was identified as a potential neurophysiological substrate for learning; but what about the substrate for reward-seeking? One possibility is that slowly varying, motivational (“tonic”) signals may prime animals to be more or less likely to initiate certain responses, but these signals would not directly trigger the movements themselves (Berke, 2018; Grace, 1991; Schultz, 2007). An alternative is that rapidly varying (“phasic”) signals may directly promote each reward-seeking episode.

The phasic signaling model has gained wide support, beginning with two studies from the Carelli laboratory reporting that subsecond dopamine fluctuations accompany food and drug reward-seeking behavior (lever pressing movements) (Phillips et al., 2003; Roitman et al., 2004). This work played an important role in challenging the narrative that dopamine only encodes RPE by suggesting that dopamine signaling represents other behavioral variables as well, including those related to rapid movements. Further work has built upon and extended this idea. Barter et al. (2015) reported that dopaminergic neuron activity was correlated to kinematic features of mouse behavior. Dodson et al. (2016) found that individual dopaminergic neurons exhibited brief changes in firing in the moments before mice initiated locomotion. Interestingly, these movement-related dynamics occurred in the absence of any overt rewards or sensory cues, suggesting that movement and RPE are distinct types of dopaminergic signals. The inability of standard RPE models to explain certain movement-related signals was formally demonstrated by Lee et al. (2019). To understand how these two distinct types of signals might coexist in the brain, Howe and Dombeck (2016) showed that dopaminergic axon activity in the striatum was topographically organized, such that dorsal striatal signals were better correlated with locomotion whereas ventral striatal signals were better correlated with rewards. This work added weight to the idea that nigrostriatal dopamine is primarily involved in movement, while mesolimbic dopamine is primarily involved in reward processing and learning (Wise, 2009). An alternative or complementary idea, presented by Mohebi et al. (2019) is that motivational signals involved in reward-seeking may be generated independently from learning-related RPE signals which are found in the somatic spiking activity of dopaminergic neurons, perhaps through the actions of striatal interneurons mediating a local motivational signal (Berke, 2018).

Additional work has identified the encoding of a wide range of psychomotor task variables pertinent to imminent or ongoing behaviors (Azcorra et al., 2023; Engelhard et al., 2019; Howe et al., 2013; Hughes et al., 2020). However, the encoding of movements does not necessarily imply a strong causal role in performing them. As in the reinforcement learning field, optogenetics has greatly facilitated exploration of dopamine’s causal contributions to movement. Howe and Dombeck (2016) also showed that optogenetically stimulating dopaminergic neuron terminals in the dorsal striatum could initiate locomotion in mice that were at rest. Interestingly, the magnitude and timing of these behavioral effects varied considerably across different test sessions. In another study, da Silva et al. (2018) found that transient dopaminergic neuron activity encodes and causally influences the vigor of movement initiation but not the production of ongoing movements. Hamid et al. (2016) showed that animals were more or less likely to initiate a reward-motivated behavioral task when dopaminergic neurons were stimulated or inhibited. They proposed that dopamine reflects a moment-to-moment value signal that affects the motivation of an animal to pursue rewards. Hamilos et al. (2021) showed that dopaminergic neurons modulate the moment-to-moment probability of initiating movements. Studies by Soares et al. (2016), Howard et al. (2017) showed that dopaminergic neuron manipulations biased the probability of selecting a particular action. Thus, an important observation is that in many cases, dopaminergic perturbations appeared to influence movement probabilistically (on a subset of trials) rather than deterministically (on all trials).

Taken together, dopaminergic stimulation is capable of rapidly influencing movement initiation, but with some caveats that will be elaborated below. Regardless, there is ample evidence that dopaminergic neurons and their projections represent more than just standard RPE signals. The signaling of rapid kinematic information, particularly the initiation of reward-motivated and spontaneous movements, appears to be a robust finding across multiple studies. The open question therefore lies in their interpretation; these signals are often taken to be functionally important for producing the movements. But as outlined in the Introduction, this interpretation hinges on an implicit assumption about the ability of phasic dopaminergic signals to rapidly influence striatal circuit activity (Figure 1). These fast modulatory effects will now be considered in detail.

## Evidence for fast dopaminergic modulation of striatal activity

A simple model of dopamine’s modulatory functions is to act on D1 or D2 receptors to raise or lower the excitability of striatal neurons, making them more or less likely to fire action potentials in response to glutamatergic input (Gerfen and Surmeier, 2011; Nicola et al., 2000). These modulatory effects were initially thought to occur relatively slowly. However, the discovery of rapidly varying dopamine signals (reviewed in the previous section) may have prompted a reassessment of the timescales involved. And certainly, studies showing that some dopaminergic neurons corelease fast-acting neurotransmitters (Stuber et al., 2010; Tritsch et al., 2012), provide at least one plausible mechanism for directly and rapidly influencing striatal spiking activity. But regardless of the specific mechanisms involved, in this section we will explore previous attempts to study the dopaminergic modulation of striatal activity and discuss some limitations of those studies that have prevented a full resolution of this issue.

Historically, early efforts to understand dopamine’s modulatory effects on striatal activity leveraged iontophoretically applied dopamine or electrical stimulation of dopaminergic pathways, and formed the basis for a number of ideas that would persist in the literature. Iontophoretic experiments involved applying current on a small glass micropipette filled with a solution that contained dopamine, causing its expulsion from the tip of the micropipette (Bloom et al., 1965; Kitai et al., 1976; Yim and Mogenson, 1982). These experiments typically occurred in anesthetized animals, and striatal neurons exhibited a mixture of increased and decreased firing upon dopamine application. However, since the amount of dopamine released was not calibrated to physiological levels, its relevance to behaviorally significant signaling events remains unclear. Another early approach came in the form of electrically stimulating the midbrain or medial forebrain bundle while recording the response of striatal neurons (Cheer et al., 2005; Connor, 1968; Gonon, 1997). But it became clear over time that this technique was not exclusively targeting dopaminergic circuits and recruited multiple neurotransmitter systems. In fact, a study by Cheer et al. (2005) found that the striatal effects of electrically stimulating the medial forebrain bundle is largely driven by GABAergic rather than dopaminergic signaling. Thus, while these experiments were influential in establishing the concept that dopaminergic input can drive changes in striatal spiking activity under certain conditions, their limitations meant that further work was needed to explore these questions.

Following these earlier approaches, other methods included pharmacological manipulation of dopaminergic neurons or receptors (Cheer et al., 2007; du Hoffmann and Nicola, 2014; Yun et al., 2004), as well as dopaminergic lesions (Albin et al., 1989; Delong, 1990). One study from du Hoffmann and Nicola (2014) involved *in vivo* electrophysiological recordings with an electrode array that was paired with a drug injection cannula, which allowed them to monitor ventral striatal activity while locally applying dopamine receptor antagonists. The experiments were carried out in rats performing a stimulus discrimination task, and the authors compared nucleus accumbens neuron firing patterns before and after drug injection. They found that both D1 and D2 receptor antagonists reduced the proportion of cells responding with excitation to a reward-paired conditioned stimulus. They also showed that these drugs reduced cued reward-seeking (lever pressing) movements. This work is important for rigorously showing that dopamine receptor signaling influences striatal information processing during behavior. However, because of the relatively slow time course of the pharmacological intervention (over several minutes), the study may not have been designed for identifying rapid and reversible electrophysiological effects on subsecond to second timescales. This issue is compounded when assessing the effects of dopamine loss after several days to months, as well as developmentally (Kim et al., 2000). While numerous studies have identified robust changes in striatal activity following neurotoxic dopaminergic lesions, it is likely many of these effects arise from the synaptic reorganization of striatal circuits unfolding over long timescales (Zhai et al., 2019). It is notable that many lesion studies were carried out to model a late stage of Parkinson’s disease and were not specifically concerned with the question of rapid modulatory effects. Thus, both pharmacological and lesion experiments lack the temporal resolution to distinguish slow from fast modulatory effects.

Of course, optogenetic manipulations address both the need for fast temporal control and cellular specificity. Some of the clearest evidence that dopaminergic neurons can rapidly alter striatal spiking came from Wang et al. (2017) who performed optogenetic stimulation of the ventral tegmental area (VTA) while performing *in vivo* electrophysiology in the ventral striatum. They found that around half of recorded striatal neurons were excited or inhibited by this stimulation within 200 ms. They went on to investigate whether these rapid electrophysiological effects arose from glutamatergic corelease by a subset of dopaminergic neurons, by repeating their experiments in a dopamine neuron-selective glutamate transporter knockout mouse. Intriguingly, though this attenuated the fast striatal responses it did not completely eliminate them, suggesting that glutamate corelease does not fully explain the rapid effect. Rapid changes in neural firing rates in the striatum were also found by Tye et al. (2012) following VTA dopaminergic neuron stimulation. They further showed that the stimulation altered striatal neuron responses to escape-related behavior. While these optogenetic experiments helped to establish that subsecond dopaminergic control of striatal activity is possible, they did not appear to consider a core issue that has been raised in recent work: if not properly calibrated, the magnitude of optogenetically evoked dopamine release could lead to supra-physiological effects.

One study that considered this issue is from Lahiri and Bevan (2020), who examined the effects of dopamine release on D1 MSNs using *ex vivo* perforated patch clamp recordings. They optogenetically stimulated dopaminergic terminals and recorded dopamine dynamics with voltammetry. The authors verified that the magnitude of these dynamics appeared similar to naturally occurring dopamine fluctuations reported *in vivo*. They found that dopaminergic stimulation produced a rapid (subsecond) and persistent (at least 10 min) PKA-dependent increase in the frequency of evoked firing of D1 MSNs. This work provides a potential mechanism for the rapid dopaminergic regulation of striatal activity. However, it is unclear if some of the experimental methods and results can be readily generalized to an *in vivo* context (McGregor and Nelson, 2020). Striatal neurons dynamically represent movements on subsecond timescales (Klaus et al., 2019). As to whether the study demonstrates that rapid dopamine signaling modulates these dynamics, the rapid effect on neural excitability indeed appears beneficial for movement initiation, while paradoxically, the persistence of this effect over several minutes appears disadvantageous for terminating movements. The long effect duration and the fact that the effect’s ceiling was reached even with minimal optogenetic stimulation (a single 2 ms pulse) suggests that the study may be more relevant for understanding the functions of slowly varying (tonic) dopamine. Indeed, MSNs *in vivo* receive multiple pulses of dopamine in a 10 min span, raising the possibility that this effect is constitutively online (Sippy and Tritsch, 2023). Nevertheless, Lahiri and Bevan’s (2020) work was important for showing that dopamine receptor second messenger cascades operated on faster timescales than previously appreciated, but their work did not preclude the need for further investigation of electrophysiological effects *in vivo*.

## Evidence for the learning primacy hypothesis of dopamine

Though the case for dopamine’s dichotomous role in learning and movement has considerable support, a more nuanced view has emerged that dopaminergic neurons only play a minor role in subsecond movement control, with their primary function being to drive associative learning (Coddington and Dudman, 2018; Lee et al., 2020; Markowitz et al., 2023; Pan et al., 2021). In subsequent discussion, we will refer to this concept as the learning primacy hypothesis of dopamine. Crucially, this view reconciles itself with the literature on movement by noting that dopamine has considerably enhanced effects on movement and striatal activity when the magnitude of evoked release is raised above physiological levels (Coddington and Dudman, 2018; Hamilos et al., 2021; Long et al., 2024).

The origin of the learning primacy hypothesis can be traced back to a paper from Coddington and Dudman (2018) that investigated the activity of dopaminergic neurons across different stages of learning a Pavlovian reward conditioning task. They found that neural activity represented a sum of RPE and movement initiation-related components. They then tested whether subsecond optogenetically evoked dopaminergic activity is sufficient to induce movements, but with an important innovation. The authors used a combined optogenetic and fiber photometry approach, enabling them to measure the magnitude of dopaminergic terminal activity being evoked. To put this magnitude into more physiological context, they calibrated the optogenetically evoked magnitude to that occurring naturally during reward presentation. They found that substantia nigra pars compacta (SNc) but not VTA dopaminergic neuron stimulation was able to induce mouse movements. But crucially, this only occurred when the magnitude of the optogenetically evoked signal was about five times larger than natural reward-evoked levels, potentially reflecting a supra-physiological response. Next, the authors confirmed that reward-matched dopaminergic neuron stimulation, in either SNc or VTA, was able to induce conditioned place preference, demonstrating that dopamine-driven learning was intact at these physiological magnitudes. This experiment was consequential for two reasons. First, it established the importance of calibrating dopaminergic circuit optogenetic manipulations against a physiological reference point (Sippy and Tritsch, 2023). Second, the study raised the prospect that dopamine’s effects on movement reported in some work may have largely been a product of optogenetic overstimulation. By extension, this would imply that dopamine’s primary role is learning while movement is not, or at least only a minor function.

In the wake of the original Coddington and Dudman study, another paper would further bolster the case for the learning primacy hypothesis. Lee et al. (2020) set out to test whether dopaminergic signaling in a Pavlovian reward conditioning task is more important for performing reward-conditioned movements (anticipatory licking), or for continuing to reinforce the cue-reward association that drives these movements. In the same animals on different sessions, they optogenetically inhibited dopaminergic neurons either during the period when animals initiate licking (before rewards) or during the period when animals evaluate the trial outcome (immediately after rewards). They found that anticipatory licking was greatly reduced with inhibition of VTA and SNc dopaminergic neurons after rewards, consistent with a strong role of these cells in learning. However, a substantially weaker change in licking was seen when inhibition took place before rewards; there was a modest effect for SNc and no significant change for VTA dopaminergic neurons. This study offered a key test for the learning primacy hypothesis, by allowing a critical direct comparison of dopaminergic neurons’ contribution to learning and movement within the same animals and behavioral task. The stronger behavioral effect of dopaminergic neuron inhibition in the post-rewards period compared to the anticipatory licking commencement period suggested that learning had primacy over movement initiation effects (with this being particularly pronounced for VTA, but also for SNc dopaminergic neurons). To further contextualize these findings, the authors went on to repeat this experiment but instead of targeting dopaminergic neurons they inhibited the secondary motor cortex, an area implicated in anticipatory movements such as licking. This time, the behavioral effects were stronger when the optogenetic inhibition occurred before rewards, implying that this cortical area is primarily involved in movement initiation rather than error-based reinforcement learning—a result diametrically opposed to the dopaminergic neuron manipulations.

The core idea expressed in the paper from Lee et al. (2020) would be affirmed and expanded on by another contemporaneous study from Pan et al. (2021). They found that physiologically calibrated dopaminergic stimulation was able to substitute for an unconditioned stimulus and maintain cue-driven approach behavior and licking behavior. They then found that dopaminergic stimulation could not induce approach behavior when substituting a cue, even after many training sessions. Thus, this work showed that dopaminergic neurons had a strong role in reinforcement while they did not appear to have a causal role in driving cue-evoked responses.

Both Pan et al. (2021), Lee et al. (2020) examined the role of dopaminergic neurons in the context of Pavlovian behavior, but this left open the question of whether the learning primacy hypothesis could be generalized to contexts without explicit rewards. A recent study from Markowitz et al. (2023) sought to do just that by monitoring dopamine signaling while optogenetically manipulating dopaminergic neurons in mice freely moving in an open field. They made the important observation that the magnitude of dopamine signals within the dorsolateral striatum to surprise rewards was similar to the magnitude of signals associated with spontaneous behavior. This suggested that reinforcement was possible in this context even if there were no explicit rewards. The authors used a closed-loop system to deliver physiologically calibrated dopaminergic terminal stimulation after detection of specific behavioral motor patterns, or syllables. Over time, this led to increased expression of the targeted behavioral syllable that persisted even after stimulation was halted. These results support a strong role for dopamine as a moment-to-moment teaching signal that shapes behavior through reinforcement, even in the absence of overt rewards.

In the above studies, it would be conceivable that physiologically calibrated levels of dopaminergic input still play a major role in rapidly shaping neural activity and that the weakness of observed behavioral effects are a reflection of inadequate behavioral measurements rather than a true reflection of dopamine’s function. Thus, a better characterization of the extent to which dopaminergic input alters subsecond *in vivo* striatal activity would be valuable for addressing this issue. Recently, we performed such experiments along with our colleagues, by combining electrophysiology, fiber photometry, and optogenetics (Long et al., 2024). By pairing a silicon probe with a photometry fiber, we were able to simultaneously record spiking activity from multiple single-units and fluorescent dopamine signaling in the striatum of awake head-fixed mice receiving unexpected rewards. An optical fiber placed above the midbrain was used to optogenetically manipulate dopaminergic neurons. We examined striatal dynamics both in the absence of, and with excess phasic dopamine using transient inhibitory and excitatory manipulations. The suppression of dopaminergic neuron activity during the reward delivery period produced a small though statistically significant change in the activity of striatal neurons. The small effect size was also observed during spontaneous firing as well as in a Pavlovian task where dopaminergic neurons were suppressed during the period of reward anticipation. The limited contribution of dopaminergic neuron signaling to rapid striatal dynamics became particularly evident when the size of these effects was compared to that of activating VTA GABAergic neurons, which rapidly and robustly altered spiking activity. Finally, we assessed the electrophysiological effects of dopaminergic neuron activation, using the simultaneously acquired photometric signals to *post hoc* calibrate the magnitude of optogenetically evoked dopamine release relative to natural reward-evoked release levels. This time, robust rapid changes in striatal spiking were observed, but crucially, this only occurred when the dopamine release magnitude exceeded around three to four times that of natural rewards. An implication of this study is that dopamine signals at ostensibly physiological levels have only a weak effect on subsecond striatal dynamics. This suggests that many of the phasic dopamine signals reported in the literature (e.g., responding to rewards, cues, and movements) may not actually play a strong role in shaping moment-to-moment striatal activity as is often implicitly assumed. By extension, this work implies that striatal spiking during movement and other rapid transient events is likely to be mainly influenced by non-dopaminergic inputs including cortical and thalamic glutamate (Lee K. et al., 2019). This study also offers a plausible means of explaining the results of Coddington and Dudman (2018), in which only supra-physiological dopamine release magnitudes meaningfully altered performance. Indeed, our optogenetic activation threshold for observing strong electrophysiological effects (∼3–4 times above reward-calibrated levels) was remarkably similar to Coddington and Dudman’s (2018) threshold for observing strong motor effects (∼5 times above reward-calibrated levels). Taken together with the behavioral studies reviewed above, this work provides some compelling evidence for the learning primacy hypothesis.

## Objections to the learning primacy hypothesis of dopamine

The first and most obvious concern is that this hypothesis may appear to downplay the literature on dopamine’s role in movement. Although work has demonstrated the potential pitfalls of overstimulating dopaminergic neurons (Coddington and Dudman, 2018), this problem largely does not apply to loss of function experiments involving inhibitory manipulations. It bears repeating that several papers have shown that optogenetically inhibiting dopaminergic neurons altered reward-guided movements, though often in a probabilistic manner (Hamid et al., 2016; Hamilos et al., 2021; Howard et al., 2017; Lee et al., 2020; Soares et al., 2016). Our electrophysiological experiments also revealed a small but statistically significant effect of inhibiting dopaminergic neurons on rapid striatal dynamics (Long et al., 2024). The question is therefore not whether a dopaminergic role in rapid motor control exists, but whether the size of this effect represents a truly vital aspect of behavior that is comparable to the demonstrably important role of dopamine in learning. One potential way to settle this issue is to contextualize dopamine’s role in movement to that of other circuits implicated in motor control, particularly those that also project to the striatum. If one could show that certain movement-related representations are enriched in dopaminergic neurons relative to other cell types, this would add weight to the idea that these neurons serve a specialized role in movement. Likewise, if one found unique or enhanced rapid behavioral effects when inhibiting dopaminergic neurons compared to inhibiting other cell types, this would present a serious challenge to the learning primacy hypothesis. In terms of the small electrophysiological effect size discussed in the previous section, showing that downstream regions can reliably read out subtle changes in striatal activity would also undermine this hypothesis.

A second objection is that this hypothesis treats dopaminergic neurons as a functionally homogeneous population, which is increasingly at odds with literature showing appreciable diversity in terms of gene expression, connections, and encoded information (Azcorra et al., 2023; Beier et al., 2015; Collins and Saunders, 2020; de Jong et al., 2022; Engelhard et al., 2019; Poulin et al., 2018). Thus, one could argue that a subpopulation of genetically or anatomically defined dopaminergic neurons may exist which predominantly serve motor functions, but that their contribution has so far been “washed out” by optogenetic perturbation methods targeting broad groups of cells. The idea that there exist genetically or anatomically distinct subsets of dopaminergic neurons for learning and movement is intriguing, but so far, the evidence supporting this is incomplete. If candidate “movement cells” are identified by means of their coding properties (Azcorra et al., 2023; Engelhard et al., 2019), we propose two crucial experiments to verify their distinct function. First, a side-by-side comparison of the effect of manipulating these cells on movement and learning behavior should be performed, perhaps by adopting the approaches of Coddington and Dudman (2018), Lee et al. (2020). Second, experiments should confirm that these cells have the capacity to effectively regulate striatal dynamics on fast timescales.

A third concern is that it may be premature to define certain dopamine release magnitudes as supra-physiological based on current calibration methods. There are a number of situations where physiological dopaminergic signaling might exceed levels induced by surprise rewards; drugs of abuse, or highly salient or aversive stimuli (Coddington and Dudman, 2019; Kutlu et al., 2021). But irrespective of limitations with the calibration method, the observation that sufficiently high levels of dopamine lead to pronounced rapid behavioral and electrophysiological effects (Coddington and Dudman, 2018; Long et al., 2024) is surprising and interesting in its own right. Regardless of whether these effects are “bugs” or “features,” their mechanisms deserve a closer look. It would be useful, for instance, to determine whether the brain’s sensitivity to rapid dopaminergic signals, and drugs acting on the dopamine system, is altered in certain disorders.

## Discussion

We have examined evidence from the literature that dopaminergic neurons primarily support a learning function and that their role in rapid movement regulation is more limited. However, a number of important objections remain that need to be further investigated. Among the objections that were discussed is the idea that dopaminergic neurons are functionally heterogeneous, potentially allowing certain genetically or anatomically distinct subpopulations to mainly support movement (Azcorra et al., 2023; Howe and Dombeck, 2016).

Nonetheless, if the learning primacy hypothesis is validated, it would have significant implications for the field. Above all, it would potentially help to move the dopamine research field forward by unifying it under a common conceptual framework. Such a unified framework would view all types of rapid dopaminergic dynamics, including movement-related activity, mainly through the lens of synaptic plasticity and reinforcement learning. Note that this would not necessarily imply the validity of standard RPE models, as further work is needed to establish whether all dopaminergic signals can be reconciled with RPE or if an alternative theory is needed (Gershman et al., 2024). Additionally, this learning-centric view has potentially important implications for understanding the pathophysiology of Parkinson’s disease that follows dopaminergic neuron degeneration. The learning primacy hypothesis predicts that the main cause of motor dysfunction is not the loss of rapid modulatory effects of dopamine on movement, but the induction of persistent synaptic changes that disrupt motor circuit function. Indeed, the prevalence of synaptic changes in Parkinson’s disease animal models is already recognized (Zhai et al., 2019). Furthermore, a recent study showed that motor function is preserved even after abolishing rapid dopaminergic fluctuations (Cai et al., 2024).

Another implication of the learning primacy hypothesis would be to spur a deeper look into the potential role of non-dopaminergic inputs to the basal ganglia in mediating movement and other fast behaviors. A plethora of brain regions projecting to the striatum appear ideally suited for motor control because they: (1) encode a variety of movement-related variables (including many of the signals which have been identified in dopaminergic neurons), and (2) release neurotransmitters which rapidly and reversibly shape downstream spiking activity.

But even if the learning primacy hypothesis fails to gain widespread acceptance, it is important for future investigations to compare and contextualize the behavioral and electrophysiological effects of dopaminergic neurons with respect to other brain areas and cell types. This approach may allow us to build a more nuanced picture of dopaminergic function that recognizes the inherently different value of knowing what these cells *can* do, versus what they can do *better* than other circuits.
